# Genetic assessment of efficacy and safety profiles of coagulation cascade proteins identifies Factors II and XI as actionable anticoagulant targets

**DOI:** 10.1093/ehjopen/oeae043

**Published:** 2024-05-27

**Authors:** Eloi Gagnon, Arnaud Girard, Jérôme Bourgault, Erik Abner, Dipender Gill, Sébastien Thériault, Marie-Claude Vohl, André Tchernof, Tõnu Esko, Patrick Mathieu, Benoit J Arsenault

**Affiliations:** Centre de recherche de l’Institut universitaire de cardiologie et de pneumologie de Québec, Y-3106, Pavillon Marguerite D'Youville, 2725 chemin Ste-Foy, Quebec, QC, Canada, G1V 4G5; Centre de recherche de l’Institut universitaire de cardiologie et de pneumologie de Québec, Y-3106, Pavillon Marguerite D'Youville, 2725 chemin Ste-Foy, Quebec, QC, Canada, G1V 4G5; Centre de recherche de l’Institut universitaire de cardiologie et de pneumologie de Québec, Y-3106, Pavillon Marguerite D'Youville, 2725 chemin Ste-Foy, Quebec, QC, Canada, G1V 4G5; Centre de recherche de l’Institut universitaire de cardiologie et de pneumologie de Québec, Y-3106, Pavillon Marguerite D'Youville, 2725 chemin Ste-Foy, Quebec, QC, Canada, G1V 4G5; Department of Epidemiology and Biostatistics, School of Public Health, Imperial College London, London, UK; Centre de recherche de l’Institut universitaire de cardiologie et de pneumologie de Québec, Y-3106, Pavillon Marguerite D'Youville, 2725 chemin Ste-Foy, Quebec, QC, Canada, G1V 4G5; Department of Molecular Biology, Medical Biochemistry and Pathology, Faculty of Medicine, Université Laval, Quebec, QC, Canada; School of Nutrition, Université Laval, Quebec, QC, Canada; Centre Nutrition, Santé et société (NUTRISS), Institut sur la nutrition et les aliments fonctionnels (INAF), Université Laval, Quebec, QC, Canada; Centre de recherche de l’Institut universitaire de cardiologie et de pneumologie de Québec, Y-3106, Pavillon Marguerite D'Youville, 2725 chemin Ste-Foy, Quebec, QC, Canada, G1V 4G5; School of Nutrition, Université Laval, Quebec, QC, Canada; Estonian Genome Center, Institute of Genomics, University of Tartu, Tartu, Estonia; Centre de recherche de l’Institut universitaire de cardiologie et de pneumologie de Québec, Y-3106, Pavillon Marguerite D'Youville, 2725 chemin Ste-Foy, Quebec, QC, Canada, G1V 4G5; Department of Surgery, Faculty of Medicine, Université Laval, Quebec, QC, Canada; Centre de recherche de l’Institut universitaire de cardiologie et de pneumologie de Québec, Y-3106, Pavillon Marguerite D'Youville, 2725 chemin Ste-Foy, Quebec, QC, Canada, G1V 4G5; Department of Medicine, Faculty of Medicine, 1050 Av. de la Médecine, Québec City, Quebec G1V 0A6, Canada

**Keywords:** Thrombin, Factor XI, Mendelian randomization, Venous thromboembolism, Bleeding, Drug target prioritization

## Abstract

**Aims:**

Anticoagulants are routinely used by millions of patients worldwide to prevent blood clots. Yet, problems with anticoagulant therapy remain, including a persistent and cumulative bleeding risk in patients undergoing prolonged anticoagulation. New safer anticoagulant targets are needed.

**Methods and results:**

To prioritize anticoagulant targets with the strongest efficacy [venous thromboembolism (VTE) prevention] and safety (low bleeding risk) profiles, we performed two-sample Mendelian randomization and genetic colocalization. We leveraged three large-scale plasma protein data sets (deCODE as discovery data set and Fenland and Atherosclerosis Risk in Communities as replication data sets] and one liver gene expression data set (Institut Universitaire de Cardiologie et de Pneumologie de Québec bariatric biobank) to evaluate evidence for a causal effect of 26 coagulation cascade proteins on VTE from a new genome-wide association meta-analysis of 44 232 VTE cases and 847 152 controls, stroke subtypes, bleeding outcomes, and parental lifespan as an overall measure of efficacy/safety ratio. A 1 SD genetically predicted reduction in F2 blood levels was associated with lower risk of VTE [odds ratio (OR) = 0.44, 95% confidence interval (CI) = 0.38–0.51, *P* = 2.6e−28] and cardioembolic stroke risk (OR = 0.55, 95% CI = 0.39–0.76, *P* = 4.2e−04) but not with bleeding (OR = 1.13, 95% CI = 0.93–1.36, *P* = 2.2e−01). Genetically predicted F11 reduction was associated with lower risk of VTE (OR = 0.61, 95% CI = 0.58–0.64, *P* = 4.1e−85) and cardioembolic stroke (OR = 0.77, 95% CI = 0.69–0.86, *P* = 4.1e−06) but not with bleeding (OR = 1.01, 95% CI = 0.95–1.08, *P* = 7.5e−01). These Mendelian randomization associations were concordant across the three blood protein data sets and the hepatic gene expression data set as well as colocalization analyses.

**Conclusion:**

These results provide strong genetic evidence that F2 and F11 may represent safe and efficacious therapeutic targets to prevent VTE and cardioembolic strokes without substantially increasing bleeding risk.

## Graphical Abstract

**Graphical Abstract:**
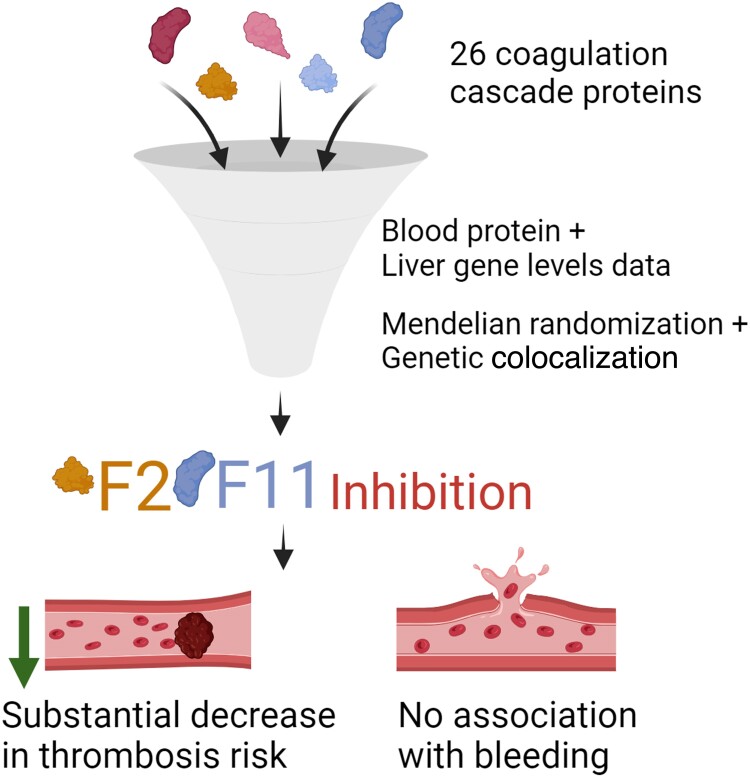

## Introduction

Conditions involving thrombotic events [i.e. ischaemic heart disease, stroke, and venous thromboembolism (VTE)] account for ∼2% of deaths worldwide, being among the leading causes of mortality.^1^ As a result, anticoagulants are routinely used in millions of patients worldwide for the treatment of different thrombotic disorders.^2^

Yet, problems with anticoagulant therapy remain, including a persistent and cumulative bleeding risk in patients undergoing prolonged anticoagulation. Meta-analyses of clinical trials have reported up to an 11% higher frequency of major bleeding in patients on warfarin or non-vitamin K antagonist oral anticoagulants compared with placebo.^3^ Anticoagulant therapies with little to no bleeding risk remain out of reach, urging the medical community to identify new targets.

All anticoagulant pharmaceutical therapies target one or more of the blood proteins involved in the coagulation cascade. Most coagulation factors are transcribed and translated into proteins in the liver and then actively secreted in the blood stream, so the liver is a preferred target for new anticoagulant therapies. Optimal anticoagulant targets need to be safe (cause low bleeding risk) and efficacious (prevent thrombosis risk). New opportunities for unravelling coagulation targets with a favourable benefit/risk ratio arise from the widespread availability of gene expression, protein, and electronic health records data from hundreds of thousands of patients. These large-scale data can be connected using the Mendelian randomization (MR) analytic paradigm to proxy the lifelong consequences of genetic perturbations of drug targets.^4^

Here, we perform two-sample MR to evaluate evidence for a causal effect of 26 coagulation cascade plasma proteins on VTE, stroke subtypes, bleeding outcomes, and parental lifespan as an overall measure of efficacy/safety ratio. We used blood protein quantitative trait loci (pQTLs) from three distinct genome-wide association studies (GWAS). We also leveraged liver expression quantitative trait loci (eQTLs) from a hepatic gene expression GWAS of 246 liver samples. These eQTLs and pQTLs were used as instruments to prioritize the therapeutic targets with the optimal efficacy (largest effect on VTE and stroke subtypes) and safety (lowest effect on bleeding outcomes) profiles.

## Methods

**Figure 1:**
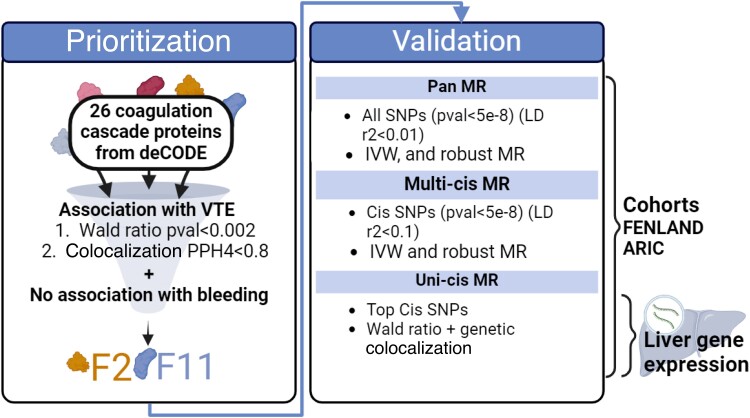
Schematic representation of the study design.

*Figure 1* displays a schema of the study design. We performed colocalization analyses and two-sample MR investigation using GWAS summary-level data. There was minimal sample overlap between data sets. All participants were of European ancestry. We harmonized the exposure and outcome data sets by aligning the effect sizes of different studies on the same effect allele. When a single nucleotide polymorphism (SNP) was not present in the outcome data sets, we used a proxy SNP (*r*^2^ > 0.6) obtained using linkage disequilibrium (LD) matrix of European samples from the 1000 Genomes Project. All GWAS used are described in Supplementary material online, *Table S1*.

### Study exposures

We included all proteins involved in the coagulation cascade as defined by PANTHER: a searchable database of gene products organized by biological function,^5^ which were measured by the SomaScan Version 4. A total of three blood pQTL data sets were used: deCODE, Atherosclerosis Risk in Communities (ARIC), and Fenland. In the deCODE population-based cohort, 4719 blood plasma protein levels were measured in 35 559 Icelanders with 4907 aptamers from SomaScan Version 4.^6^ The aptamer levels were adjusted for age and sex, and the resulting residuals were inverse rank normal transform prior to GWAS. We extracted associations between 27 million SNPs measured by Illumina SNP chips and 26 blood coagulation proteins measured using 33 SOMAmers. In the ARIC population-based cohort, 4657 plasma proteins were measured in 7213 European Americans using 4657 aptamers from SomaScan Version 4.^7^ The relative abundance of SOMAmers was adjusted in a linear regression model including probabilistic estimation of expression residuals (PEER) factors and the covariates sex, age, study site, and 10 ancestry-based principal components. We extracted associations between cis-SNPs measured with Infinium Multi-Ethnic Global BeadChip array (Illumina, GenomeStudio) and 25 blood coagulation proteins measured using 32 SOMAmers. In this data set, only summary statistics on cis-acting variants, defined as the transcription starting ±500 kb, is available. In the Fenland population-based cohort, 4775 plasma proteins were measured in 10 708 European-descent participants with 4978 aptamers using the SomaScan v4 assay.^8^ The aptamers levels were adjusted for age, sex, the first 10 principal genetic components, and test site, and the resulting residuals were inverse rank normal transform prior to GWAS. We extracted association between SNPs and 26 blood coagulation proteins measured using 33 SOMAmers. We also used liver gene expression of coagulation cascade proteins as study exposures from the Institut Universitaire de Cardiologie et de Pneumologie de Québec Obesity Biobank (https://iucpq.qc.ca/en/research/platforms/biobank). Liver samples were obtained by incisional biopsy of left lobe and immediately snapped frozen. Read counts and transcript per million were produced with RNA-SeQC v2.4.2.^9^ Measures were adjusted for sex, age, the top 10 genetics principal components, and 15 PEER factors. We mapped liver eQTLs using samples obtained from 246 participants that passed genotyping and RNA sequencing quality controls, as previously described.^10^ The deCODE data set was used for primary analysis because it provides more statistical power given the sample size, whereas the other data sets were used for replication analyses. We now include this information in the abstract.

### Study outcomes

For VTE, we performed a GWAS meta-analysis of three cohorts: the UK Biobank, the Estonian Biobank, and FinnGen. We performed two novel GWAS for VTE in the Estonian Biobank and the UK Biobank (data application number 25205). In the UK Biobank and the Estonian Biobank, VTE cases were defined as self-reports confirmed by a trained medical professional of deep vein thrombosis, pulmonary embolism, or VTE or according to electronic health record codes (ICD-10: I80.2, I80.2, I82.2, I26.0, and I26.9, OPCS-4: L79.1 and L90.2).^11^ Participants identified with cases of other forms of thrombosis (ICD-10: I81, I80.0, I80.3, I80.8, and I80.9), known coagulation defects (D68) or Budd–Chiari syndrome (I82.0) were excluded from the analysis. Participants without these codes were identified as controls. We included a total of 17 200 European cases and 387 625 controls from the UK Biobank and 12 569 European cases and 164 827 control in the Estonian Biobank. We used the SAIGE (Scalable and Accurate Implementation of Generalized Mixed Models)^12^ algorithm to perform GWAS in the UK and Estonian Biobanks. This method adjusts for population-based factors such as sample relatedness while still maintaining maximum statistical power. We used sex, age, and the 10 first ancestry-based genetic principal components as covariates in the analysis. We then meta-analysed these results with VTE GWAS summary statistics obtained from the FinnGen’s data freeze 7.^13^ This GWAS included 14 454 cases and 387 625 controls. FinnGen GWAS includes summary statistics for 16 million genetic markers genotyped using the Illumina or Affymetrix arrays. The meta-analysis was completed using the METAL algorithm, using a fixed-effect inverse variance weighted meta-analysis.^14^ Overall, the VTE GWAS meta-analysis includes 9 572 513 SNPs with minor allele frequency ≥0.01 in 44 223 cases and 847 152 controls.

We used GWAS summary statistics from a meta-analysis of the International Stroke Genetics Consortium consortium and the UK Biobank totalling 73 652 cases of stroke 1 234 808 controls of European ancestry.^15^ Cases were defined with clinical diagnosis of strokes (62 100 cases of ischaemic stroke, 10 804 cases of cardioembolic stroke, 6399 cases of large artery stroke, 6811 cases of small vessel stroke, and 1 234 808 controls). The GWAS were adjusted for age, sex, principal components of population stratification, and study-specific covariates when needed.

For bleeding outcomes, we used GWAS summary statistics from a GWAS of the FinnGen population-based cohort totalling >309 000 individuals of European ancestry.^13^ Cases were established with electronic health record ICD10 codes (GI bleeding ICD10 = K92[0–2]; intracranial haemorrhage ICD10 = ‘I9_SAH|I9_ICH’; bleeding ICD10 = ‘D3_HAEMORRHAGCIRGUANTICO|H7_CONJUHAEMOR|H7_RETINAHAEMORR|H7_VITRHAEMORR|H7_CHORHAEMORRHAGE|ST19_EPIDU_HAEMORRHAGE|I9_INTRACRA|I9_OTHINTRACRA|ST19_TRAUMAT_SUBDU_HAEMORRHAGE|ST19_TRAUMAT_SUBAR_HAEMORRHAGE|R18_HAEMORRHAGE_RESPI_PASSA|K11_GIBLEEDING’). The GWAS was performed in SAIGE (v.0.35.8.8) and adjusted for sex, age, genotyping batch, and 10 first principal genetic components as covariates.

We used GWAS summary statistics for two parents’ survival from a meta-analysis of the UK Biobank and the LifeGen consortium of 26 population cohorts (*n* = 1 012 240; all of European ancestry).^16^ The outcome was defined as parental survival in a Cox model. Mother and father survival information was combined assuming the effects were the same in men and women.

### Mendelian randomization

We applied three types of MR analysis to assess the causal effect of coagulation factors on VTE, stroke, bleeding outcomes, and parental lifespan. Cis-acting pQTLs (close to the gene of interest) are more specific instruments, suppressing or upregulating the expression of a gene, whereas trans pQTLs (distal to the gene) may operate via more complex mechanisms, making them more likely to be pleiotropic. ‘cis-MR’ refers to an instrument selection strategy using SNPs in the gene region only, by opposition to ‘pan MR’ that would refer to selecting SNPs throughout the genome. First, we used a uni-cis-MR framework. We selected the top SNP with the smallest *P*-value in a 1 Mb window of the coagulation cascade gene regions. Mendelian randomization estimates on each protein and outcomes were obtained with the Wald ratio. The Wald ratio is calculated by dividing the SNP-outcome effect by the SNP-exposure effect. To assess instrument strength, we used the *F*-statistic,^17^ and to quantify the variance explained, we used the *r^2^* value.^18^ An issue that can arise when performing uni-cis MR analysis is the potential inclusion of functional variants altering the structure of the protein and creating epitope-binding artefacts. Epitope-binding artefacts refer to the scenario where a structural change in protein decreases the aptamer affinity for this protein, resulting in a biased association with the genetic variant and the measured protein levels. To evaluate the possibility of epitope-binding artefacts, we annotated all genetic instruments using the variant effect predictor and removed instruments with evidence of being altering variant. We evaluated reverse causality by performing the Steiger test. The Steiger test provides a *P*-value under the null hypothesis that the difference in variance explained is null.^19^ We performed the Steiger test to identify variants with evidence of a stronger association with the outcome than with the exposure.

We also used a multi-cis MR approach where we selected multiple independent cis-acting variants as genetic instruments. Multi-cis MR analysis, that is, using multiple genetic variants, allows performing robust MR analyses to evaluate robustness to horizontal pleiotropy in the causal estimates.^20^ For this analysis, we included as genetic instruments all cis-acting SNP in a 1 Mb window around the gene region independently (LD clumping = *r*^2^ < 0.1) associated at *P* < 5e−08 with protein levels. Third, we used pan MR analysis (all cis- and trans-acting variants). We selected independent (*r*^2^ < 0.01) genome-wide significant SNPs (*P* < 5e−08) from all regions of the genome and performed MR analysis. The inclusion of trans-acting pQTLs increases the number of independent genetic instruments, thus increasing power, allowing the use of robust analysis and the quantification of heterogeneity (Cochran’s Q) to assess the validity of the MR assumptions but can introduce pleiotropy.

For multi-cis and pan MR analysis, we performed the inverse variance weighted method with multiplicative random effects with a standard error correction for under dispersion as primary MR analysis.^21^ We also perform three different robust MR analyses (the MR Egger,^22^ the contamination mixture,^23^ the weighted median, and the MR PRESSO^24^) to explore if their estimate substantially differed from the primary MR results. These methods, their assumptions, and their statistical property have been extensively reviewed elsewhere.^25^

### Genetic colocalization

Spurious association can occur because of LD. LD bias occurs when the true causal SNP of the exposure and the outcomes are distinct, but in LD, causing a false positive MR association. To evaluate bias due to LD, we performed genetic colocalization analysis. We evaluated the posterior probability that both the protein or the RNA and the outcomes shared a single variant using a Bayesian model implemented in the *coloc* R package.^26^ We included all variants in a 1 Mb window of the gene regions. We used the default priors for the analysis. The prior probability a SNP is associated with Trait 1 (p1) was set 1e−4, the prior probability a SNP is associated with Trait 2 (p2) was set to 1e−4, and the prior probability a SNP is associated with both traits (p12) was set to 1e−5. We used a posterior probability H4 > 0.50 as a threshold for evidence of colocalization signifying that colocalization is more likely than any other scenario combined.^27^

## Results

### Venous thromboembolism genome-wide association meta-analysis

We performed a new VTE GWAS meta-analysis including two new GWAS in the Estonian Biobank and UK Biobank and an existing GWAS from FinnGen. The meta-analysis includes 44 223 cases identified through electronic healh records and self-identifications confirmed by trained nurses and 847 152 controls. This meta-analysis identified 3,017 SNPs significantly associated with VTE (*P* < 5e−08) spread throughout the genome at 77 independent risk loci (LD *r^2^* < 0.01; see Supplementary material online, *Figures S1* and *S2*), all of which have been previously identified.^28^ These loci were then annotated using the nearest gene. Notably, two of these loci were mapped to F2 and three other loci were mapped to F11 as the nearest gene.

### Estimated effects of plasma coagulation proteins using uni-cis

As discovery methods, we first performed uni-cis MR where we used the variant with the lowest *P*-value in the cis-acting region ±1 Mb as instruments and calculated the effect with a Wald ratio. Most proteins were measured in the three cohorts (deCODE, ARIC, and Fenland). Across all study cohorts, all proteins had at least one top SNP with an *F*-statistic > 10 indicating sufficient strength of instruments. The top SNPs of the PLAT, PLAUR, and KNG1 genes were variant altering the amino acids sequence. Epitope-binding artefact was unlikely for the remaining 23 proteins. To evaluate reverse causality, we performed the Steiger test, where genetic instruments that explain more variance in the outcome than the exposure are tagged. No genetic instruments were tagged by Steiger test indicating that reverse causality was unlikely.

**Figure 2:**
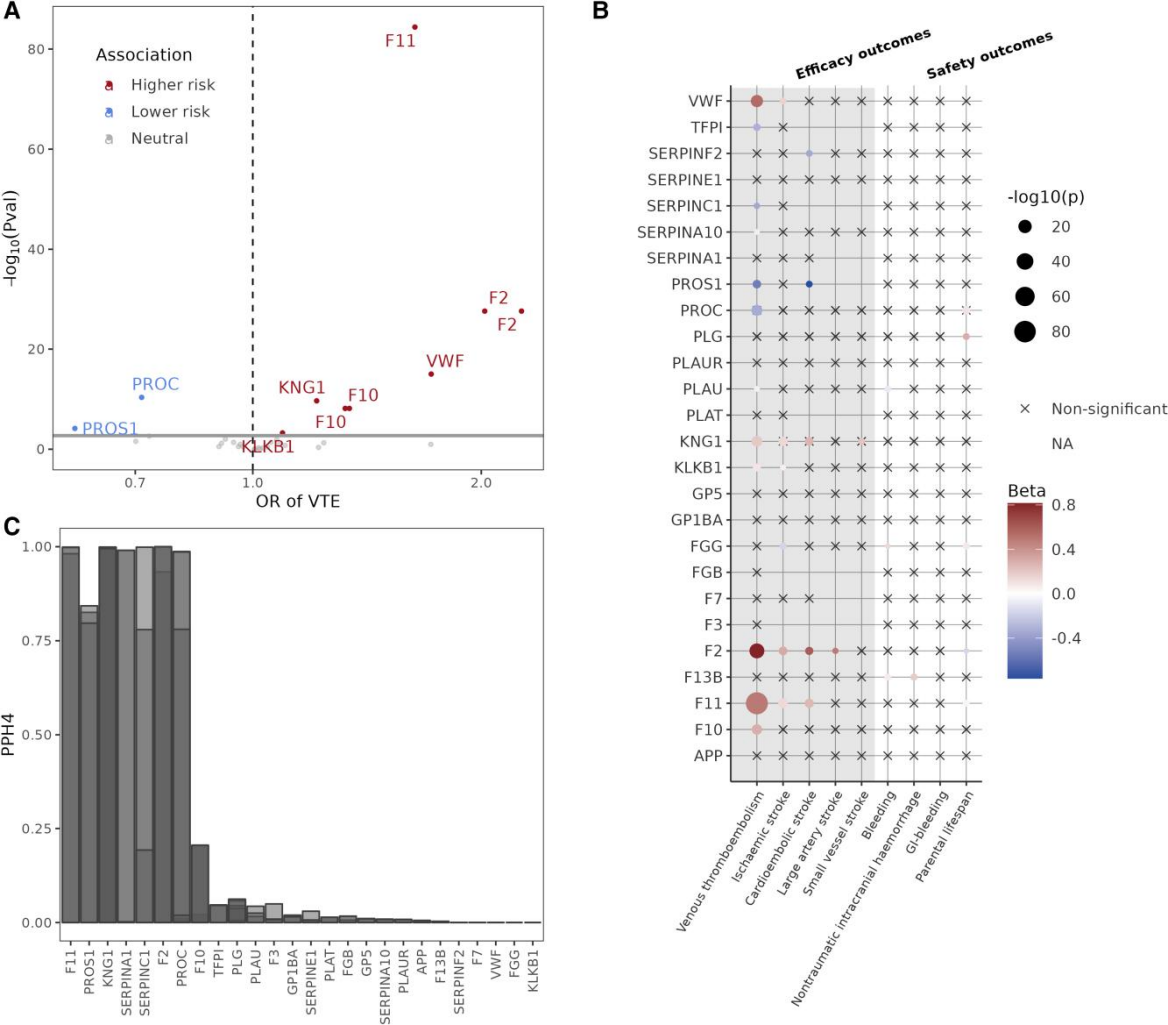
Genetically predicted effect of 26 coagulation pathway blood protein levels on safety and efficacy outcomes. (*A*) Volcano plot of the association of all proteins with venous thromboembolism risk in deCODE. (*B*) Balloon plot presenting effect sizes and posterior probability of hypothesis 4 in deCODE non-available associations stem from a lack of overlapping single nucleotide polymorphisms or proxies (*r*^2^ > 0.6) between exposure and outcome data resulting in no overlapping single nucleotide polymorphism in the harmonized data set. Associations at *P* > 0.05 are depicted with crosses. (*C*) Posterior probability of genetic colocalization (posterior probability of hypothesis 4) between deCODE, Atherosclerosis Risk in Communities, and Fenland proteins and venous thromboembolism.

**Figure 3:**
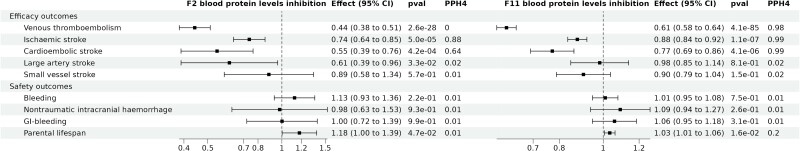
Genetically predicted reductions in blood F2 and F11 levels on safety and efficacy outcomes in a uni-cis Mendelian randomization analysis. Effect of 1 SD decrease (inhibition) in F2 or F11 blood protein levels in deCODE on efficacy and safety outcomes. PPH4 stands for ‘posterior probability of hypothesis 4’ as calculated with the coloc method, the posterior probability that two traits share a common variant.

Using the uni-cis MR framework, among all the 26 coagulation cascade blood proteins studied, F2 and F11 most strongly affected VTE and ischaemic stroke (*Figure 2*). In deCODE, genetically predicted reductions in F2 blood levels (mimicking the effect of F2 inhibitors) as measured with the aptamer 5316_54 was associated with lower VTE risk [odds ratio (OR) per 1 SD lower F2 = 0.44, 95% confidence interval (CI) = 0.38–0.51, *P* = 2.6e−28] and cardioembolic stroke (OR = 0.55, 95% CI = 0.39–0.76, *P* = 4.2e−04), with a comparatively smaller increased risk in bleeding (OR = 1.13 95%, CI = 0.93–1.36, *P* = 2.2e−01). Similarly, genetically predicted reductions in F11 blood levels (mimicking the effect of F11 inhibitors) were associated with lower risk of VTE (OR = 0.61, 95% CI = 0.58–0.64, *P* = 4.1e−85) and cardioembolic stroke (OR = 0.77, 95% CI = 0.69–0.86, *P* = 4.1e−06) but were not associated with bleeding (OR = 1.01, 95% CI = 0.95–1.08, *P* = 7.5e−01; *Figure 3*). Mendelian randomization results were similar in ARIC and Fenland data sets (see Supplementary material online, *Figure S3*).

Bayesian genetic colocalization revealed strong probability of shared variants between F2/F11 and VTE/cardioembolic stroke, indicating that confounding by LD was unlikely. Across samples and aptamers, the mean posterior probability of hypothesis 4 (PPH4) for F2 and VTE was 0.66, for F2 and cardioembolic stroke was 0.61, for F11 and VTE was 0.99, and for F11 and cardioembolic stroke was 0.99. There was no evidence of colocalization between F2 and bleeding and F11 and bleeding (PPH4 < 0.02; see Supplementary material online, *Figure S4* and *Table S2*).

### Validation using multi-cis and pan Mendelian randomization

Multi-cis MR analyses provided concordant evidence that F2 and F11 inhibition was associated with decreased risk of VTE and stroke and not associated with bleeding (see Supplementary material online, *Figure S5*). For multi-cis MR, we selected all genome-wide significant SNPs in the cis region ± 1 Mb and pruned them with a correlation threshold of *r^2^* < 0.1. Consistent with what was obtained using the uni-cis MR analysis, genetically predicted reductions in F2 blood levels were associated with decreased VTE risk (OR = 0.46, 95% CI = 0.41–0.52, *P* = 3.5e−33) and cardioembolic stroke risk (OR = 0.58, 95% CI = 0.44–0.78, *P* = 2.8e−04), with a comparatively smaller increase risk in bleeding (OR = 1.14, 95% CI = 0.99–1.31, *P* = 6.2e−02). Similarly, genetically predicted F11 reduction were associated with lower risk of VTE (OR = 0.66, 95% CI = 0.63–0.69, *P* = 3.9e−64) and cardioembolic stroke (OR = 0.84, 95% CI = 0.79–0.88, *P* = 8.5e−10) but not with bleeding (OR = 0.99, 95% CI = 0.96–1.02, *P* = 6.5e−01; *Figure 3*). Mendelian randomization results were similar using robust MR analyses and similar in ARIC and Fenland data sets (see Supplementary material online, *Figure S6* and *Table S3*). Altogether, multi-cis MR provides convergent evidence that F2 and F11 inhibition is associated with a decrease in VTE and cardioembolic stroke risk but not associated with bleeding risk.

**Figure 4:**
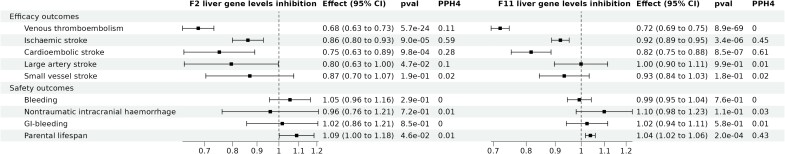
Genetically predicted reductions in liver expression of *F2* and *F11* on safety and efficacy outcomes in a uni-cis Mendelian randomization analysis. Effect of 1 SD decrease (inhibition) in F2 or F11 hepatic gene expression levels on efficacy and safety outcomes. PPH4 stands for ‘posterior probability of hypothesis 4’ as calculated with the coloc method, the posterior probability that two traits share a common variant.

Pan MR analysis revealed a strong effect of F2 and F11 on VTE and cardioembolic stroke (*Figure 4*). Trans-acting pQTLs are more likely to act on protein levels via pleiotropic pathways. However, their inclusion in MR analysis can increase the variance explained and prevent reliance on a single genetic region. We therefore included all genome-wide significant SNPs throughout the genome clumped at *r*^2^ < 0.01 in a 1 Mb window region and performed a pan MR analysis. The inverse variance weighted method was used as primary MR analysis, and other robust MR analyses (MR PRESSO, contamination mixture, and weighted median) were used as sensitivity analyses. In the deCODE sample, using pan MR analyses, genetically predicted reduction in F2 blood levels was associated with lower VTE risk (OR = 0.56, 95% CI = 0.42–0.75, *P* = 9.5e−05) and cardioembolic stroke (OR = 0.52, 95% CI = 0.40–0.67, *P* = 2.7e−07) risk but did not associate with bleeding (OR = 0.98, 95% CI = 0.89–1.08, *P* = 6.9e−01). Similarly, genetically predicted F11 reduction was associated with lower risk of VTE (OR = 0.79, 95% CI = 0.74–0.84, *P* = 7.9e−13) and cardioembolic stroke (OR = 0.86, 95% CI = 0.81–0.91, *P* = 4.9e−07) but not bleeding (OR = 1.00, 95% CI = 0.96–1.03, *P* = 9.0e−01; see Supplementary material online, *Table S4*). For F11, these effects were significant and directionally consistent across all robust MR methods indicating robustness to pleiotropy and replicated in the Fenland cohort (see Supplementary material online, *Figure S7*). For the association between F2 and VTE, the contamination mixture method did not reach significance in deCODE but did in Fenland. For the effect of F2 on VTE and cardioembolic stroke, several robust MR methods did not reach statistical significance for the aptamer 4157_2 but did with the aptamer 5316_54. Violation of the exclusion restriction assumption therefore cannot be ruled out for the aptamer 4157_2. We could not perform pan MR analysis in the ARIC cohort, because only cis summary statistics are publicly available.

### Impact of liver gene levels of F2 and F11 on efficacy and safety outcomes

F2 and F11 are specifically expressed in the liver and are then secreted in the bloodstream. We tested the hypothesis that hepatic expression of F2 and F11 might also be causally associated with efficacy and safety outcomes. To test this hypothesis, we used the same uni-cis MR approach as with the blood proteins but used instead hepatic gene expression levels (Methods). Because the sample size was lower for this data set, the top SNPs had larger *P*-value (1.1e−05 for F2 and 3.0e−12 for F11). Variance explained was nevertheless high (*r^2^* = 0.08 for *F2* and 0.18 for *F11*) and the instrument was strong (*F*-statistic of 20 for *F2* and 55 for *F11*). Using the Wald ratio formula, genetically predicted reduction in *F2* hepatic gene level was associated with lower VTE risk (OR per 1 SD lower *F2* = 0.68, 95% CI = 0.63–0.73, *P* = 5.7e−24), lower cardioembolic stroke risk (OR = 0.75, 95% CI = 0.63–0.89, *P* = 9.8e−04), and increased lifespan (0.08 years 95% CI = 0.00–0.17, *P* = 4.6e−02) but did not associate with bleeding (OR = 1.05, 95% CI = 0.96–1.16, *P* = 2.9e−01). Genetically predicted reductions in liver *F11* gene levels were associated with lower VTE risk (OR = 0.72, 95% CI = 0.69–0.75, *P* = 8.9e−69), lower cardioembolic stroke risk (OR = 0.82, 95% CI = 0.75–0.88, *P* = 8.5e−07), and increased lifespan (0.04 years 95% CI = 0.02–0.06, *P* = 2.0e−04) but did not associate with bleeding (OR = 0.99, 95% CI = 0.95–1.04, *P* = 7.6e−01) nor any other bleeding outcomes. Overall, the effect size of hepatic gene expression was smaller than that of blood proteins. This is expected since blood protein levels is a more proximal effector of blood coagulation and presumably not all hepatic mRNA is transcribed into proteins.

Colocalization yielded low-to-moderate probability of shared causal variant between F2/F11 and VTE/ischaemic stroke. The PPH4 for F2 and VTE was 0.11, for F2 and cardioembolic stroke was 0.28, for F11 and VTE was 0.00, and for F11 and cardioembolic stroke was 0.61 (see Supplementary material online, *Figure S4*). The colocalization between liver F11 and VTE yielded a PPH4 of 0 and a PPH3 of 0.99, contrasting with the colocalization between blood F11 protein levels and VTE, which yielded a PPH4 of 0.99 and a PPH3 of 0 (see Supplementary material online, *Table S2*). The difference in colocalization results is most likely due to differential sample size. Overall, these analyses support that reduction of hepatic gene levels of F2 and F11 are associated with lower VTE and ischaemic stroke but not bleeding risk.

## Discussion

To prioritize new targets for anticoagulant therapy, we performed a new VTE GWAS and examined evidence for a causal effect of 26 coagulation cascade plasma proteins on VTE, stroke subtypes, bleeding outcomes, and parental lifespan. Using a comprehensive MR strategy, we found that genetically predicted inhibition of F2 and F11 were associated with lower risk of VTE and ischaemic stroke, without substantially increasing bleeding risk. These associations were concordant across three large blood pQTL data sets, one liver eQTL data set, and across several robust MR methods. Altogether, if substantiated by randomized clinical trials, these results provide strong genetic evidence that F2 and F11 may represent safe and efficacious therapeutic targets to prevent VTE and cardioembolic stroke without substantial increase in bleeding risk.

Our MR results on F2 are concordant with results of clinical trials comparing direct thrombin inhibitors with placebo.^29,30^ Direct thrombin inhibitors specifically inhibit the intrinsic activity of the F2 gene product: the protein thrombin. The administration of the direct thrombin inhibitors dabigatran at a dose of 150 mg twice daily strongly reduced the risk of VTE (hazard ratio, 0.08; 95% CI 0.02–0.25) but increased the risk of major or clinically relevant bleeding (hazard ratio, 2.92; 95% CI 1.52–5.60) when compared with placebo in patients with VTE.^30^ Similarly, Dabigatran 110 mg vs. placebo twice daily lowered the 2-year risk of major vascular complications (hazard ratio, 0.72; 95% CI 0.55–0.93) but increased risk of minor bleeding (hazard ratio, 1.64; 95% CI 1.25–2.15), but not major bleeding, among patients who had myocardial injury after non-cardiac surgery.^29^ Our MR results similarly outline this advantageous efficacy/safety ratio.

Preclinical and clinical investigations support a role of *F11* inhibition in decreasing VTE risk with mild increase in bleeding risk. First, *F11*-deficient mice do not appear to have haemostatic defects. Indeed, bleeding time in mice lacking *F11* is comparable with wild type mice after injury to the tail.^31–33^ One study, however, found that *F11*-deficient mice display a moderate haemostatic defect in a saphenous vein bleeding model.^31^ But this result failed to replicate in another study where saphenous vein bleeding time was the same in *F11^−/−^* mice compared with wild-type mice.^34^ Second, patients with severe *F11* deficiency have a 4.68 lower risk of VTE, a 8.56 lower risk of ischaemic stroke but the same incidence of myocardial infarction compared with individuals with normal *F11* function.^35,36^  *F11*-deficient individuals are not at higher risk of spontaneous bleeding, however risk of bleeding following trauma or surgery increases.^37^ Third, a previous MR investigation, using a different instrument selection strategy, provided evidence that lower levels of F11 protect against VTE and cardioembolic stroke but does not increase bleeding risk.^38^ The latter study, however, did not test other coagulation cascade proteins or liver gene expression, so they could not prioritize targets or assess the effect of perturbing liver gene expression. A similar study, assessing the effects of 1151 proteins on VTE, prioritized Factor XI and Factor II among the targets with the highest effect on VTE.^39^ However, bleeding risk was not assessed, so the safety of these targets was unknown. Our results extend those of previous MR investigations,^39–41^ by showing that not only F2 and F11 may represent efficacious target for VTE prevention, but they are also likely to be safe. Finally, several types of *F11* inhibitors are currently being tested in humans and have demonstrated good efficacy and safety. Published Phase 1 trials do not report any safety concerns for *F11* inhibitors.^42^ F11 inhibition with liver-targeted antisense oligonucleotides (*F11*-ASO),^43^ small molecules (milvexian),^44^ or monoclonal antibodies (abelacimab)^45^ in patients undergoing total knee surgery were significantly superior to enoxaparin, in terms of rates of VTE.

RNA therapeutics targeting hepatic *F2* or *F11* are promising anticoagulant targets. RNA therapeutics such as ASOs or small interfering RNAs are used to silence gene expression by complementary base pairing with messenger RNA. The liver is an organ of choice for RNA therapeutics delivery. Antisense oligonucleotides have been chemically engineered to bind asialoglycoprotein receptors, which are present on the surface of hepatocytes but not on other cell types or tissues. Antisense oligonucleotides for *F11* mRNA are currently under development.^43^ In the *F11*-ASO TKA study, 300 patients undergoing total knee surgery were randomized to receive one of two doses of an ASO against *F11* (IONIS F11-LRx 200 or 300 mg) or 40 mg of enoxaparin once daily.^43^ At 200 mg, *F11*-ASO was non-inferior (27%), while the 300 mg was superior (4%) to enoxaparin (30%; *P* < 0.001) for preventing a composite outcome of symptomatic VTE or asymptomatic deep VTE. Rates of bleeding were inferior in the *F11*-ASO groups (both 3%) than in the enoxaparin group (8%).^43^ Antisense oligonucleotide targeting possesses a convenient mode of delivery, an efficient reversal strategy, and good efficacy (prevent thrombosis) and safety (low bleeding risk) profile. Antisense oligonucleotide therapy decreases *F11* levels so it could be rapidly reversed with an injection of *F11*. Although ASO therapies are currently only delivered using subcutaneous injection, their long half-life allows conveniently for weekly or potentially monthly self-administration. Our results from genetically predicted liver expression of the genes encoding *F2* and *F11* support further investigation into the role of RNA interfering therapies in the prevention of VTE.

The main strength of this study is the triangulation of evidence using several data sets and several MR methodologies providing convergent results. The main limitation of the study is that all anticoagulant proteins could not be evaluated. There are at least 40 proteins involved in the coagulation pathway,^5^ but the Somascan V4 only assesses 26 of them. It is possible that other coagulation cascade proteins may have more optimal efficacy or safety profile, but they could not be tested in our MR framework.

## Conclusions

These results support that inhibition of F2 and F11 hepatic gene expression and/or blood protein levels may reduce the risk of thrombosis without substantially increasing the risk of bleeding. *F2* is an established target for anticoagulation. *F11* is an emerging target of three different anticoagulant therapies under development with various inhibition strategies. These findings provide genetic support for therapies either targeting hepatic gene expression or blood protein levels of *F2* or *F11*, potentially opening a new era of anticoagulant therapies.

## Supplementary Material

oeae043_Supplementary_Data

## Data Availability

Code for this manuscript can be found at https://github.com/LaboArsenault/F2F11. The GWAS summary statistics for VTE can be retrieved on the GWAS catalogue https://www.ebi.ac.uk/gwas/. All other genome-wide summary statistics used in this study are in the public domain.
