# Supplementary material for: Genetic assessment of efficacy and safety profiles of coagulation cascade proteins identifies Factors II and XI as actionable anticoagulant targets
Source: Eur Heart J Open. 2024 May 27;4(3):oeae043. doi: 10.1093/ehjopen/oeae043 (PMC11200102; doi:10.1093/ehjopen/oeae043)
Supplement: oeae043_Supplementary_Data [file oeae043_supplementary_data.zip › Supplementary_Figures.pdf]

Supplementary Figures

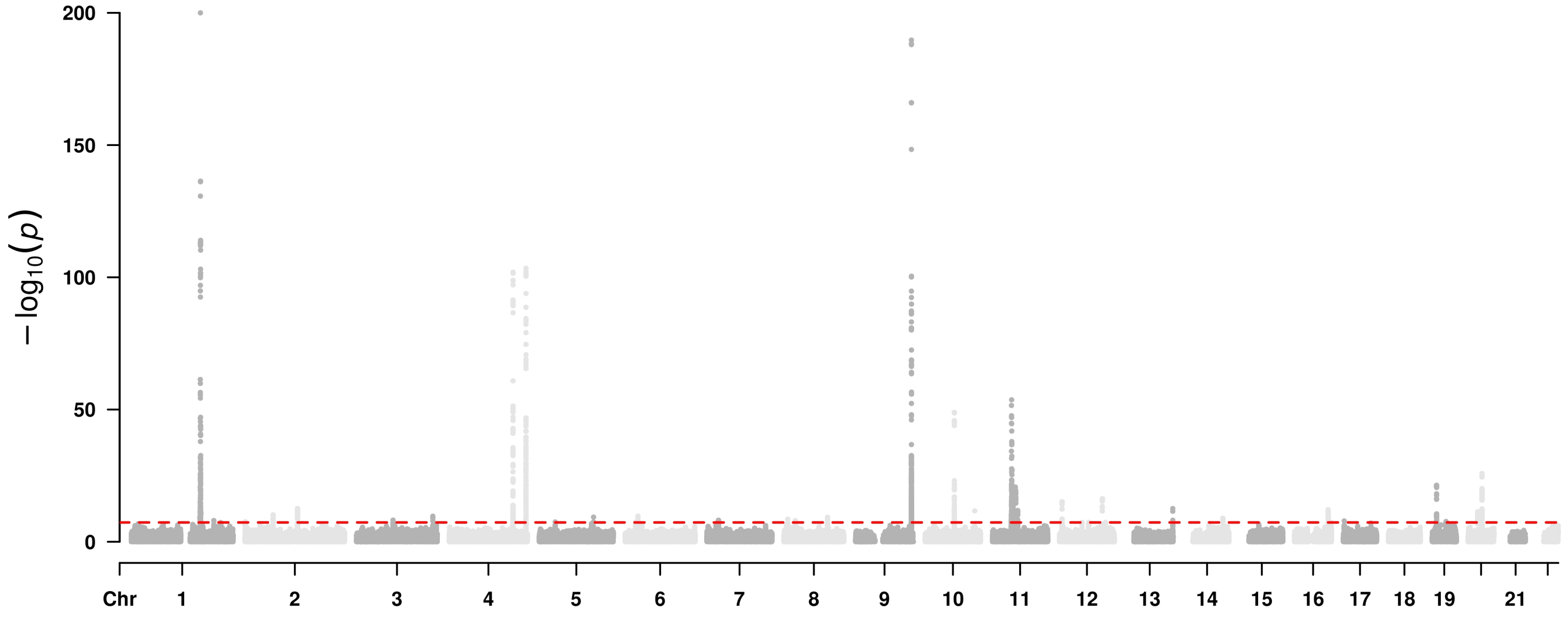

Supplementary Figure 1. Manhattan plot of venous tromboembolism. The x axis represents the chromosomal position. The y axis represents $-\log_{10}(p)$. Each point represent one single nucleotide polymorphism.

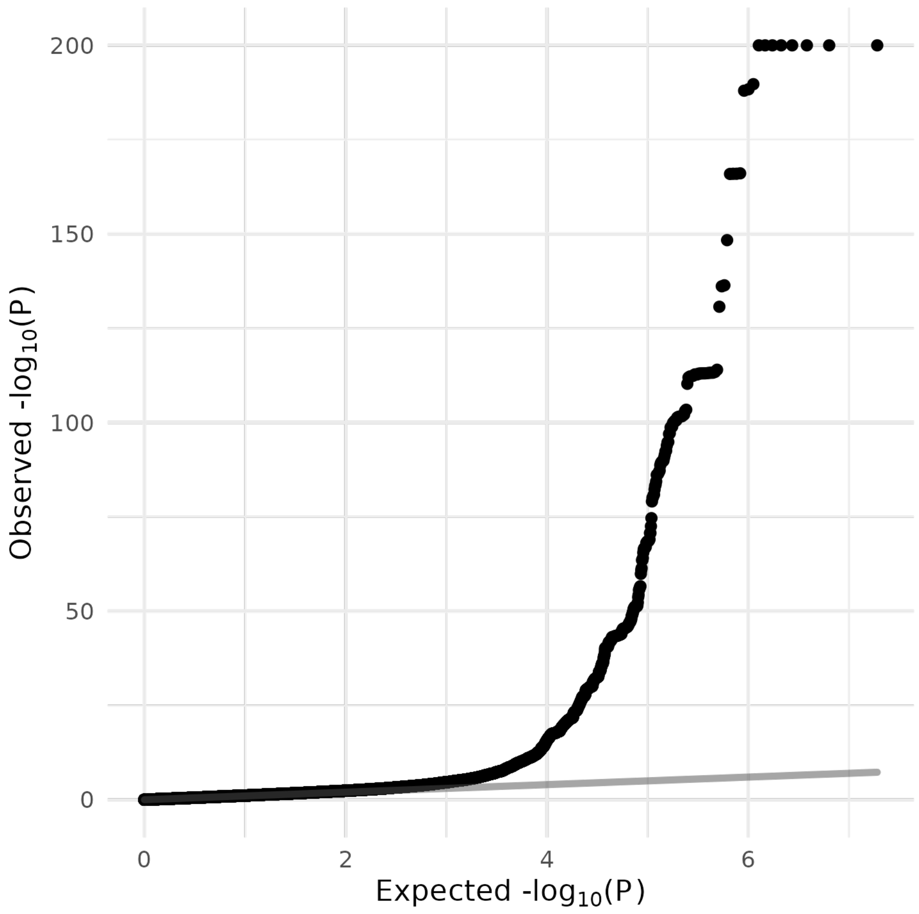

Supplementary Figure 2. QQ plot of the venous tromboembolism genome-wide association study. The x axis represents the expected $-\log_{10}(p)$. The y axis represents the observer $-\log_{10}(p)$. The shape of the plotted distribution is concordant with some true positive hits.

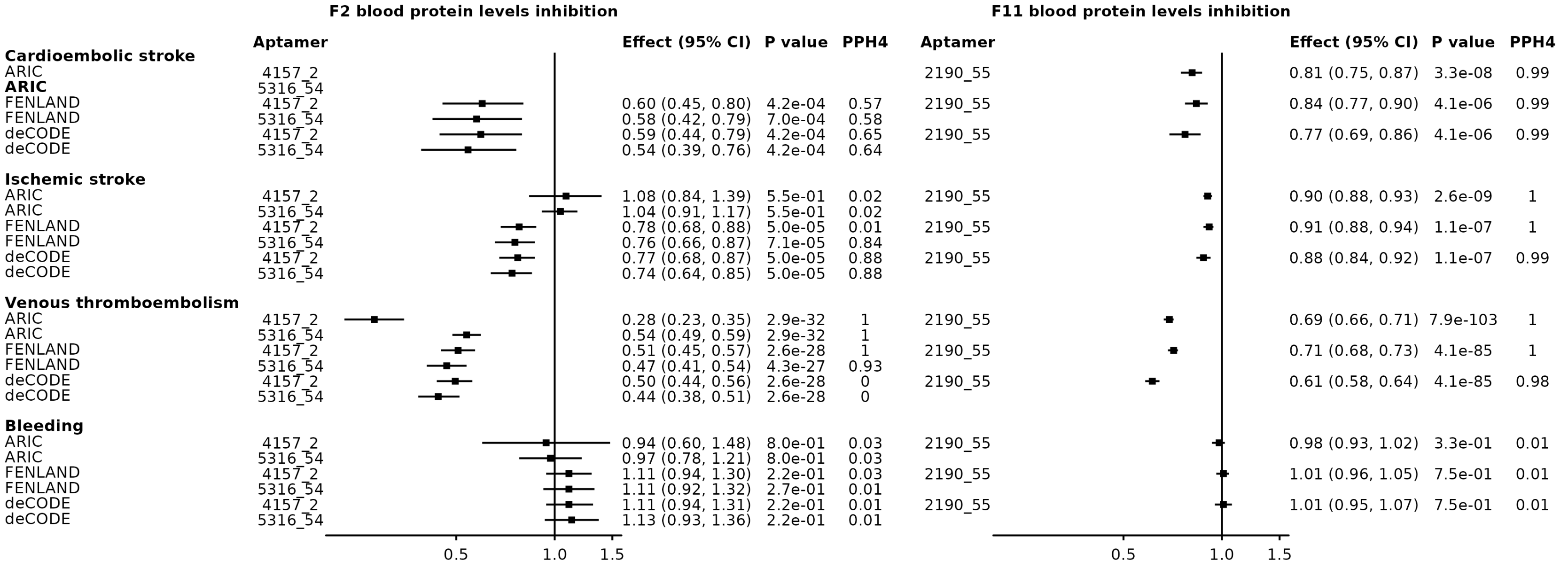

Supplementary Figure 3. Uni-cis MR method. Effect of 1 standard deviation decrease (inhibition) in F2 or F11 blood protein levels accross three different samples on efficacy and safety outcomes.

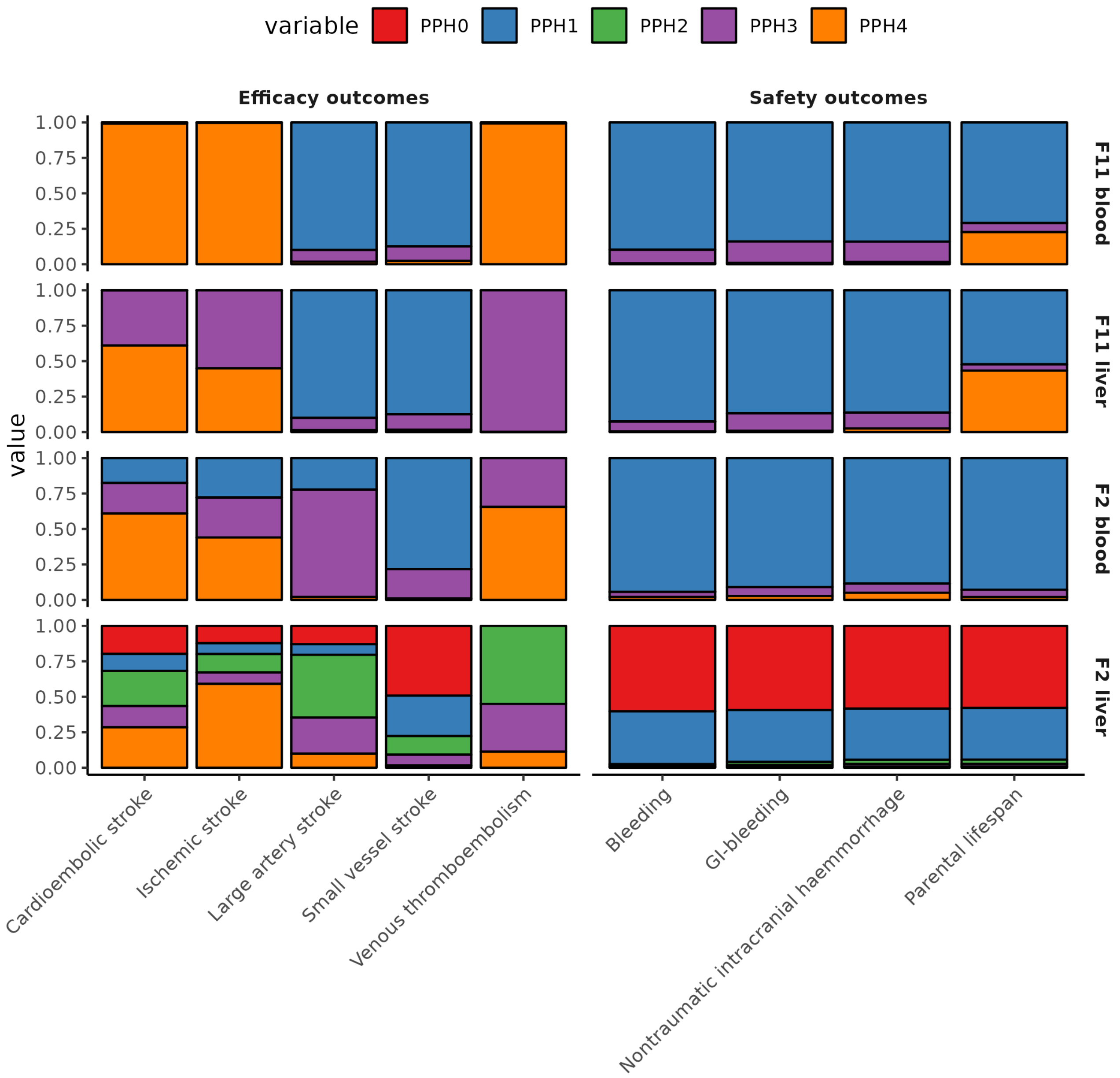

Supplementary Figure 4. Colocalisation results. Hepatic gene expression/blood preotein levels as trait 1. Efficacy and safety outcomes as trait 2. PPH = posterior probability of hypothesis. PPH0 = probbility of no causal variants for both traits; PPH1 = probability of causal variants for trait 1, but not trait 2; PPH2 = probability of causal variant for trait 2, but not trait 1; PPh3 = probability of causal variants for both traits, but distinct; PPH4 = probablity of shared causal variant.

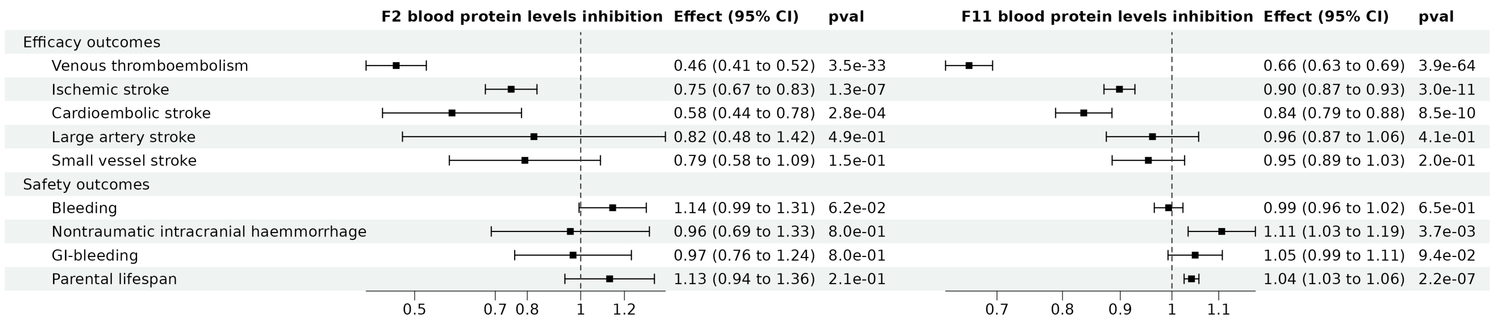

Supplementary Figure 5. Genetically predicted reductions in blood F2 and F11 levels on safety and efficacy outcomes in a multicis MR analysis. Effect of 1 standard deviation decrease (inhibition) in F2 or F11 blood protein levels measured in deCODE on efficacy and safety outcomes

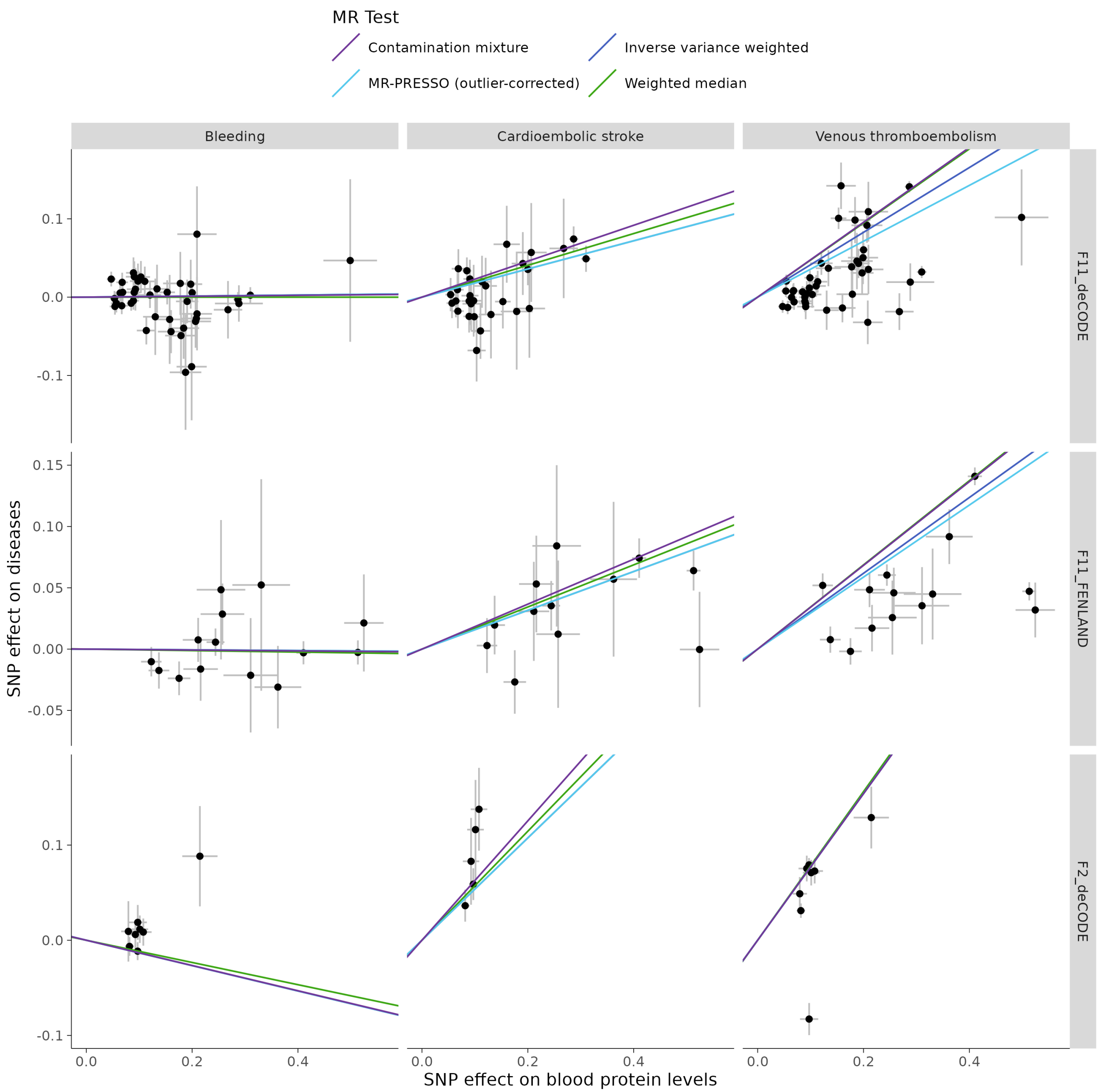

Supplementary Figure 6. Muli-cis MR method. Effect of of F2 and F11 blood protein levels accross samples on efficacy and safety outcomes. Robust MR results are displayed.

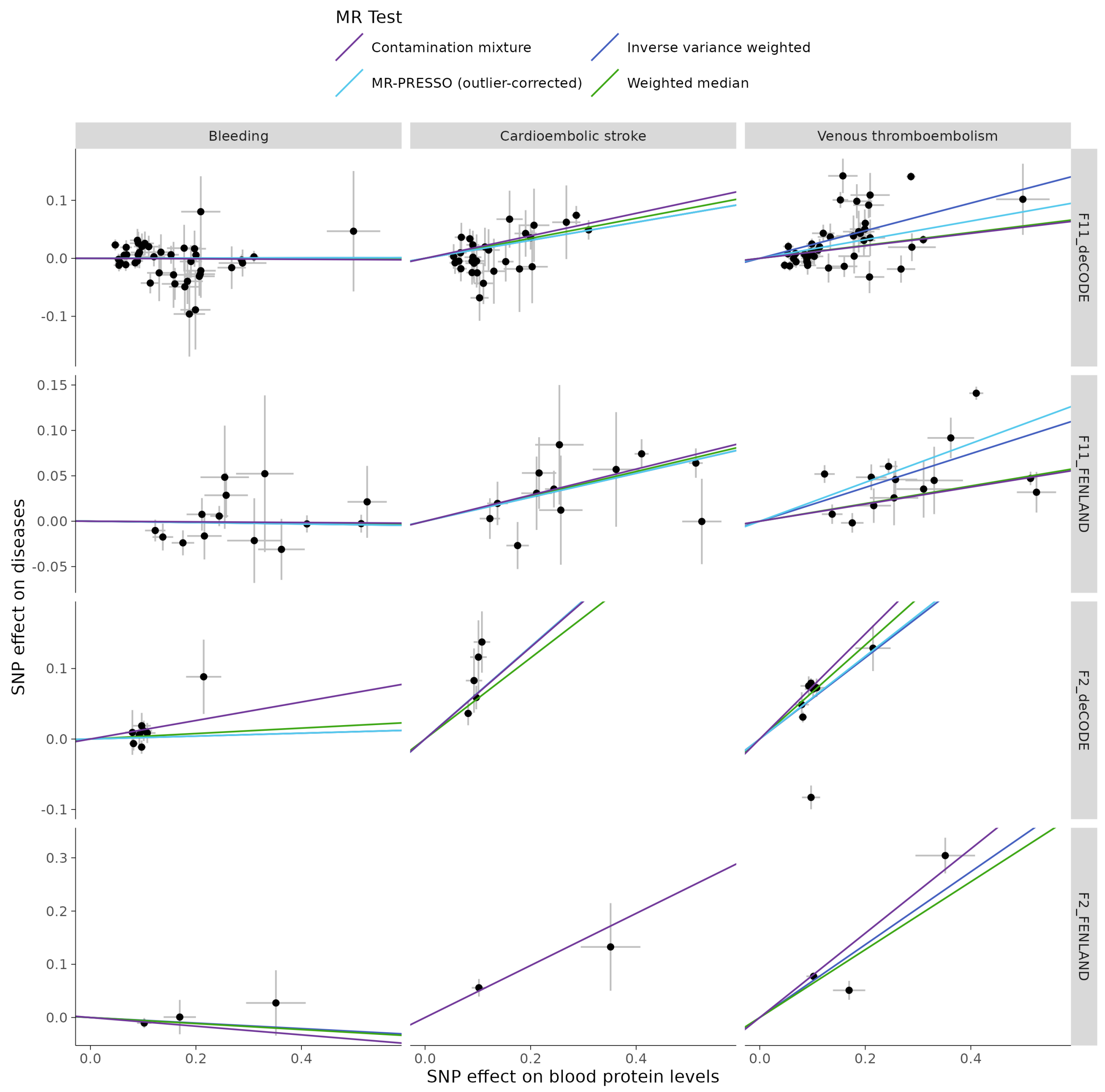

Supplementary Figure 7. Genetically predicted reductions in blood F2 and F11 levels on safety and efficacy outcomes in a pan MR analysis. Scatter plot of the association between F2 and F11 in deCODE sample on ischemic stroke, venous thromboembolism, and bleeding. Inverse variance weighted methods and robust MR analyses are presented.
